# Factors associated with facility childbirth and skilled birth attendance in Migori County, Kenya and the effect of Lwala Community Alliance intervention: a cross-sectional assessment from the 2019 and 2021 Lwala household surveys

**DOI:** 10.3389/fgwh.2024.1426264

**Published:** 2024-09-19

**Authors:** Christina Hope Lefebvre, Joseph R. Starnes, Aleksandra Jakubowski, Alyn Omondi, Janet Manyala, Jane Wamae, Ash Rogers, Sandra Mudhune, Vincent Okoth, Vincent Were, Julius Mbeya, Samantha V. Yap, Philip Omondi, Willys Ochieng, Tom Odhong, Carren Siele, Richard Wamai

**Affiliations:** ^1^ Department of Biology, Northeastern University, Boston, MA, United States; ^2^ Department of Pediatrics, Division of Pediatric Cardiology, Vanderbilt University Medical Center, Nashville, TN, United States; ^3^ Lwala Community Alliance, Rongo, Kenya; ^4^ Department of Health Sciences, Northeastern University, Boston, MA, United States; ^5^ Department of Economics, Northeastern University, Boston, MA, United States; ^6^ Department of Research, Adaptive Model for Research and Empowerment of Communities, Kisumu, Kenya; ^7^ Department of Cultures, Societies, and Global Studies, Northeastern University, Boston, MA, United States; ^8^ Department of Health, County Government of Migori, Migori, Kenya; ^9^ Nigerian Institute of Medical Research, Lagos, Nigeria; ^10^ African Centre for Community Investment in Health, Chemolingot, Kenya; ^11^ Integrated Initiative for Global Health, Northeastern University, Boston, MA, United States

**Keywords:** skilled birth attendance, facility childbirth, Lwala Community Alliance, community health worker, community health organization, access to healthcare, healthcare disparities

## Abstract

**Background:**

Despite evidence of the beneficial effects of skilled birth attendance (SBA) on maternal health and childbirth outcomes, there are disparities in access across counties in Kenya. These include Migori County which has historically recorded high maternal mortality rates. In 2007, the Lwala Community Alliance was founded to improve health outcomes in this county. The objective of this study is to provide a baseline status of facility childbirth and SBA in Migori and to characterize the effect of Lwala intervention on these outcomes.

**Methods:**

A cross-sectional household survey was designed for a 10-year study to evaluate the effectiveness of Lwala initiatives. The 2019 and 2021 household surveys were conducted in Lwala intervention wards and in comparison wards with sample sizes of 3,846 and 5,928 mothers, respectively. The survey captured demographic, health, and socioeconomic data at each household, data on SBA and facility childbirth, and explanatory variables. A generalized linear model was used to determine factors associated with SBA. A secondary trend analysis was conducted to determine change over time in the explanatory variables and SBA. To determine the change in SBA rate due to Lwala intervention, controlling for background temporal trends, a difference-in-differences (DiD) model compared SBA rates in intervention wards and comparison wards.

**Results:**

SBA increased in all surveyed wards and across all explanatory variables from 2019 to 2021. The DiD analysis showed that the SBA rate increased more in Lwala intervention wards than in comparison wards (Adjusted Prevalence Rate Ratio 1.05, *p* < 0.001, 95%CI 1.03–1.08). The 2021 survey found the highest rates of both facility childbirths (97.9%, 95%CI 96.5–98.7) and SBA (98.2%, 95%CI 97.0–99.0) in North Kamagambo, the oldest ward of Lwala intervention. Higher educational status, four or more ANC visits, marriage/cohabitation, and wealth were significantly associated with increased SBA.

**Conclusions:**

We provide the first quasi-experimental evidence that Lwala interventions are significantly improving SBA which may inform related initiatives in similar settings. The household-survey data provides a baseline for continued evaluation of Lwala programs, and the breakdown by ward allows for development of specific programmatic targets.

## Introduction

In recent years, coordinated efforts between the World Health Organization (WHO) and the United Nations (UN) have made progress in reducing maternal mortality, but the global burden remains high with an estimated 287,000 maternal deaths in 2020 (1, 2). Data indicate that progress plateaued between 2016 and 2020, with an annual global average reduction in MMR of approximately zero, as compared to 2.7% per year from 2000 to 2015 (2). The global MMR remains over three times greater than the 2030 Sustainable Development Goal's (SDG) target of 70 maternal deaths per 100,000 live births (2). Furthermore, the majority of the global burden is attributed to low- and middle-income countries; in 2020, 70% of maternal deaths occurred in sub-Saharan Africa (1, 2). Within sub-Saharan Africa, Kenya has made progress in promoting safe pregnancy and childbirth, reporting a decline in MMR from 432 deaths per 100,000 live births in 2010 to 342 deaths per 100,000 live births in 2017 (3). This MMR remains nearly five times the SDG target for 2030. As of 2015, 15 of 47 counties in Kenya held a disproportionate 98% of this maternal mortality burden (4).

Despite Kenya's progress in healthcare service delivery, significant disparities in access to skilled birth attendance (SBA) remain (5). In 2021, almost 90% of maternal deaths in Kenya could be attributed to insufficient quality of care (5). Additionally, only one third of public health facilities in Kenya had the capacity to provide the seven critical interventions to resolve basic obstetric and neonatal health emergencies (5). Three in every four maternal deaths in Kenya were associated with direct, preventable causes, including postpartum hemorrhage, hypertensive disorders, infections, and complications during childbirth (5). Providing accessible, timely clinical care could significantly mitigate antenatal care (ANC) concerns and reduce maternal mortality rates (6, 7).

Studies in sub-Saharan Africa have established that SBA is a critical factor in improving newborn survival and decreasing maternal mortality (8–10). Survey data from 29 countries in sub-Saharan Africa collected between 2010 and 2018 demonstrate 63% of births assisted by skilled health personnel (11). The 2022 SDG report cites an increase to 70% SBA in sub-Saharan Africa, but this percentage is still far below the global average of 86% coverage (2). The COVID-19 pandemic may have further disrupted progress (2, 12). Additionally, the low density of skilled health personnel in the region creates challenges for increasing SBA rates (11, 13–15). Data from 2014 to 2021 demonstrate that the densities of medical doctors and of nursing and midwifery personnel in sub-Saharan Africa are 2.3 and 12.6, respectively, per 10,000 population, as compared to 39.4 medical doctors per 10,000 population in Europe and 152.1 midwifery and nursing staff per 10,000 population in North America (2). The WHO estimates that approximately 45 skilled health personnel per 10,000 population are necessary to accomplish sufficient skilled birth coverage (16). Low quality of care may also impede utilization of existing SBA (14, 15, 17, 18).

Among counties in Kenya with high maternal mortality is Migori. The County has a population of 1.1 million and is located in western Kenya in the former Nyanza province (19). Migori County has historically cited poor health metrics, with only 53.4% of births delivered by skilled health personnel in 2014, as compared to the national average of 61.8% (20). This trend was reversed when Migori surpassed the national average (89.3%) in the 2022 Demographic and Health Survey, at 92.6% (21). In 2014, only 53.3% of births in Migori County were delivered in a health facility as compared to the national average of 61.2% (20). This value has also improved to 89.2% as compared to the 2022 national average of 82.3% (21).

The Lwala Community Alliance (Lwala) was founded in 2007 to promote health and wellbeing in Migori County (4). The first initiative of the organization was to establish a rural primary healthcare facility which provides inpatient, outpatient, maternal, HIV, and other holistic primary health care services. In the last decade, Lwala has partnered with the Ministry of Health to implement a community-led health model. This model includes strengthening communities to launch local health initiatives and participate in governance of the health system, supporting government health facilities to improve service quality for maternal and child health, and training, paying and equipping community health workers (CHWs) to bring care to every home. The novel CHW program recruits all active traditional birth attendants (TBAs) in a region of interest to the broader CHW program, integrating them into the formal health system (4). TBAs are deeply trusted by their neighbors and are the main competitors to skilled health personnel. Lwala's strategy is to transform TBAs into champions for SBA as well as other key maternal and child health services, including ANC, postpartum care, immunizations, and family planning. This partnership with existing structures promotes community trust in formal health interventions, rather than competing to provide healthcare. Subsequently, Lwala partnered with the Migori County government in 2022 to pass the landmark Community Health Services Act to promote CHW professionalization and community-level healthcare leadership (22).

A repeated cross-sectional household survey was designed to track progress associated with Lwala initiatives and to identify potential areas for improvement (23). This study aims to characterize the impact of Lwala intervention on SBA and facility childbirth and to provide baseline data for future program evaluation.

## Methods

### Study design

A cross-sectional household survey was designed to assess various health metrics in wards with and without Lwala programming over a 10 year period. The survey health indicators include childhood mortality, vaccination coverage, ANC, contraceptive prevalence, and SBA. The sample size was established to detect a 10% difference in a health metric over time with 80% power for each of these wards. This study utilizes data from the 2019 and 2021 cross-sectional surveys conducted in Lwala intervention wards and comparison wards. The objective is to outline the current status of facility childbirth and SBA in Migori, which may inform future programmatic efforts, and to characterize the effects of Lwala interventions on rates of SBA.

### Study setting

**Figure 1:**
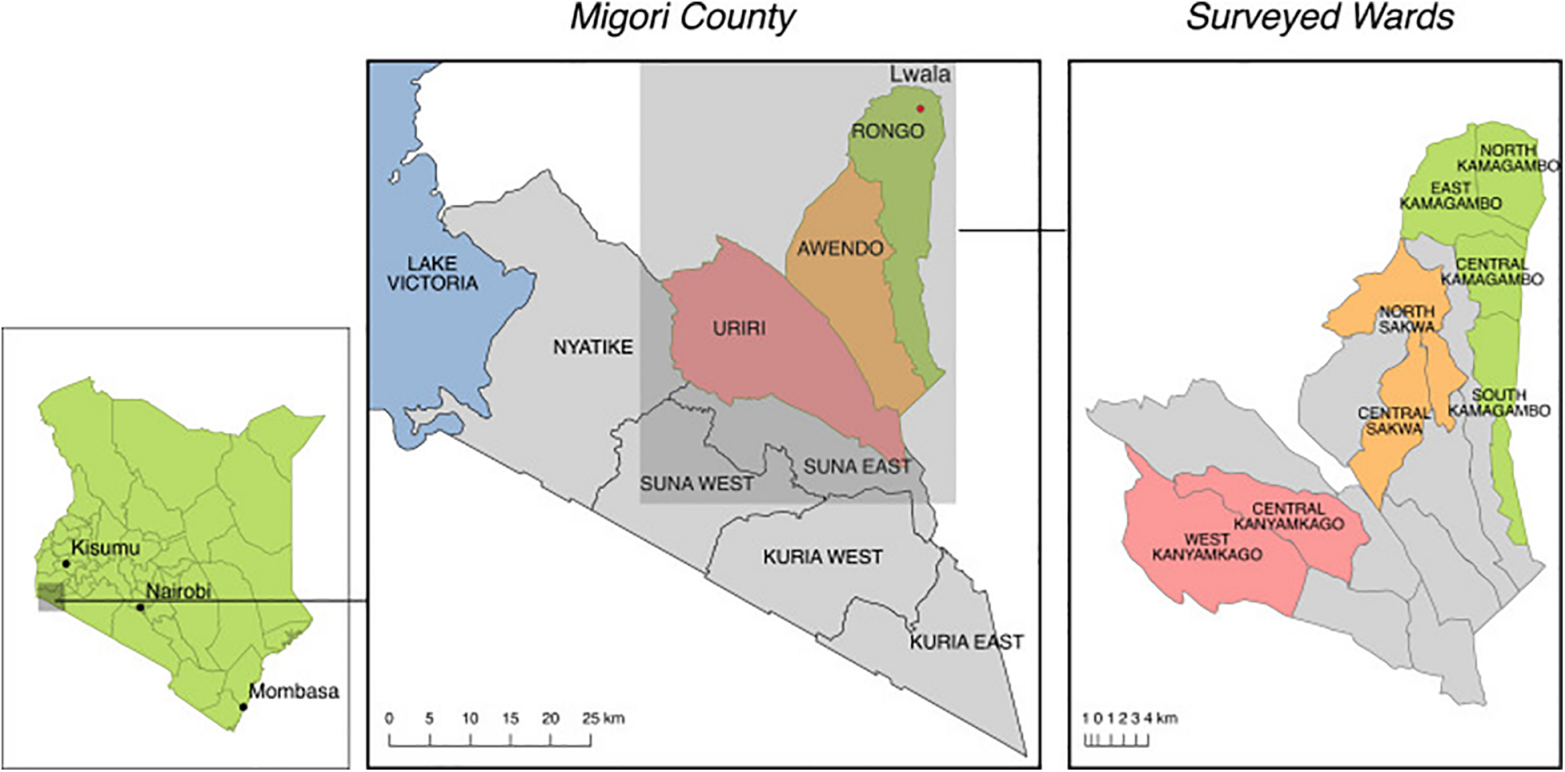
Lwala programming began in Rongo sub-county (green) and has expanded to parts of Awendo sub-county (orange) and the entirety of Rongo. Programs were not yet active in Central Kamagambo, North Sakwa, or Central Sakwa in 2021. Comparison wards from Uriri sub-county (red) include West Kanyamkago and Central Kanyamkago. (23).

This study was domiciled in three sub-counties of Migori County, Kenya (Table 1). Migori County borders Lake Victoria in western Kenya (Figure 1) (23). The county has eight sub-counties, including Rongo sub-county where Lwala is based. Lwala was founded in North Kamagambo, one of the four administrative wards of Rongo sub-county. It has since expanded to cover the entire Rongo sub-county, including North, East, South, and Central Kamagambo (NK, EK, SK, and CK). Lwala interventions include the establishment of rural healthcare facilities, promotion of an inclusive, community-led health model, and creation of government partnerships to aid in the professionalization of CHWs.

**Table 1:** Implementation date of Lwala intervention and survey timepoints by ward.

Sub-county	Ward	Lwala intervention implementation	2017^a^	2019	2021
Rongo	North Kamagambo	2007	X	X	X
	East Kamagambo	2018		X	X
	South Kamagambo	2019		X	X
	Central Kamagambo	2021 (post-survey)		X	X
Awendo	North Sakwa	2022			X
	Central Sakwa	2022			X
Uriri	Central Kanyamkago	Comparison		X	X
	West Kanyamkago	Comparison		X	X

^a^ Survey was conducted as part of previous work.

^b^ “X” marks survey timepoint.

Data used in this study include three wards where Lwala was operating prior to the 2021 survey (NK, EK, SK), three wards where Lwala interventions were implemented after the 2021 survey (CK of Rongo sub-county and North Sakwa and Central Sakwa of Awendo sub-county), and two comparison wards of Uriri sub-county (Central Kanyamkago and West Kanyamkago) (Table 1).

### Sampling and survey

The sampling and survey methodologies are described in depth in a prior manuscript (23). In summary, a hybrid household sampling procedure was derived from the World Health Organization Expanded Programme of Immunization (EPI) (24, 25). The approach utilized Geographic Information System (GIS) technology to divide each area into grid squares with a precise center point. Survey teams began their day at this center point and used the spin-the-bottle technique to randomly select households (23). The bias introduced by the original EPI methodology was minimized by the use of an arbitrary starting location as opposed to a town center (26). Each grid square had a set number of seven households to be surveyed. There was a deliberate bias to sample more women with children below the age of five years due to Lwala's programmatic focus on maternal and child health. Therefore, out of the seven households to be surveyed per grid square, five were required to include women with children below the age of five years. The other two households sampled in each grid square had male household heads or women with children above the age of five years. The people surveyed at each household were the head of the household. The 2019 survey sample size was 3,846 mothers, and the 2021 survey sample size was 5,928 mothers.

The survey was administered through a customized questionnaire on a Research Electronic Data Capture (REDCap) tool (27, 28). The survey questions were derived from validated tools for measuring the relevant health metrics (20). Specifically, it included a complete birth history of all children born to the respondent or their spouse, including the birth location and the profession of the person who assisted with the birth of the child (23). Demographic, health, and socioeconomic data were captured about the respondent and household (23).

### Statistical analysis

Data cleaning and analysis were conducted using Stata version 15 (StataCorp LP, College Station, TX). The outcome variables were facility childbirth and skilled birth attendance. The most recent birth occurring within five years prior to the survey was considered for this analysis. Facility childbirth was coded as “Yes” for respondents who said that they delivered at a public/private health facility or Lwala Community Alliance hospital and “No” if the respondent delivered at home. Skilled Birth Attendance was coded as “Yes” if the respondent was assisted by a clinician/nurse/doctor/trained or certified midwife and “No” if the respondent was not assisted by any skilled health personnel or was assisted by a traditional birth attendant/relative/friend.

The explanatory variables consisted of place of residence (North Kamagambo, East Kamagambo, Central Kamagambo, South Kamagambo, Central Kanyamkago, West Kanyamkago, North Sakwa, and Central Sakwa), mother's age at birth (≤20, 21–30, 31–40, 41–50, >50) (where minimum age was 13 and maximum age was 69), education level (primary or less, secondary or more), marital status (married/cohabiting, unmarried), religion (Catholic, Seventh-Day Adventist, Protestant, Roho Church, other), ANC visits (<4, ≥4), parity (1, 2–3, 4+), community health worker (CHW) visitation in the last three months (yes/no), year (2019/2021), and wealth tertile (poor, middle, rich).

Descriptive analysis of both 2019 and 2021 data was conducted where SBA and facility childbirth frequencies, percentages, and prevalence confidence intervals were reported. To determine factors associated with skilled birth attendance, a generalized linear model using a Poisson distribution with log-link function was used to estimate prevalence rate ratios, also commonly known as prevalence odds ratios. As only 0.9% of 2019 facility childbirths and 0.4% of 2021 facility childbirths did not utilize SBA, the regression analysis was only run with SBA data. The facility childbirths without SBA likely occurred during off hours when no skilled health personnel were present. Prevalence rate ratios were preferred over logistic regression because the outcomes of interest were common (had high prevalence), and therefore, the latter method would overestimate the odds ratio. Both bivariate and multivariate analyses were performed. Adjusted Prevalence Rate Ratios (APR), 95% Confidence Interval, and *p*-value were reported. A *p*-value of <0.05 was considered statistically significant.

To further examine temporal changes in the explanatory variables and SBA, a secondary trend analysis was conducted. Trend Prevalence Ratios (adjusted), 95% Confidence Interval, and *p*-value were reported. Only variables where data was available for both 2019 and 2021 were included in these analyses. Mother's age at birth, CHW visitation, and parity were not available in the 2019 dataset, as these were added between surveys based on programmatic interest. A separate regression analysis was conducted with the 2021 dataset for just these variables. None of the three factors was significantly associated with SBA, so the variables were dropped from the larger regression analysis. To distinguish the change in SBA rate due to Lwala intervention from background temporal changes, a difference-in-differences (DiD) model was used. The DiD model compared the change over time in the outcome of interest (SBA rate) between the intervention wards and the comparison wards. The DiD methodology quantifies the additional difference in a given metric with intervention over a background increase (or decrease) in that metric in the population (29). The design allows for comment about causation and not only association as in simpler modeling strategies. This model utilized a Poisson regression and included an interaction term to account for timing of survey observations at all intervention wards. This interaction term of intervention wards and post-intervention is the coefficient of interest. This coefficient measures the change in SBA in wards with Lwala intervention as compared to those without, adjusted for covariates, including education level, marital status, religion, ANC, and wealth.

### Ethical approval

The study was approved by the Ethics and Scientific Review Committee of AMREF Health Africa (AMREF-ESRC P452/2018) and the Institutional Review Board of Northeastern University (IRB #: 20-09-18). Written consent was obtained from all survey participants.

## Results

### Demographics

A total of 3846 mothers were surveyed in 2019, and 5,928 mothers were surveyed in 2021 (Table 2). The birth age group with the greatest percentage of respondents in 2021 was 21–30 years old (57%). In 2021, most respondents had been visited by a CHW (87.9%). In both 2019 and 2021, most respondents had attended at least four ANC visits (66.8% and 79.3%, respectively).

**Table 2:** Demographics of respondents from the 2019 and 2021 datasets.

Variables	2019 (*n* = 3,846)		2021 (*n* = 5,928)	
	Frequency	%	Frequency	%
Place of residence				
NK	203	5.3	730	12.3
EK	601	15.6	748	12.6
CK	1,064	27.7	778	13.1
SK	1,014	26.4	736	12.4
CKK	484	12.6	727	12.3
WK	480	12.5	770	13.0
NS	–	–	758	12.8
CS	–	–	681	11.5
Mother's age at birth^a^				
≤20	–	–	1,212	20.5
21–30	–	–	3,380	57
31–40	–	–	1,052	17.8
41–50	–	–	194	3.3
>50	–	–	90	1.5
Education level				
Primary or less	2,264	58.9	3,127	52.8
Secondary or more	1,578	41.1	2,801	47.3
Marital status				
Married/Cohabiting	2,895	58.9	5,239	88.4
Unmarried	1,578	41.1	689	11.6
Religion				
Catholic	581	15.3	926	15.6
Seventh-Day Adventist	1,515	39.9	2,395	40.4
Protestant	875	23.1	1,215	20.5
Roho Church	635	16.8	999	16.9
Other	184	4.9	393	6.6
ANC visits				
<4	1,104	33.2	1,229	20.7
≥4	2,222	66.8	4,699	79.3
CHW visitation				
Yes	–	–	2,130	87.9
No	–	–	293	12.1
Wealth tertile				
Poor	1,272	33.4	1,967	33.3
Middle	1,271	33.3	1,974	33.5
Rich	1,271	33.3	1,960	33.2
Parity				
1	–	–	1,848	32.1
2–3	–	–	2,616	45.5
4+	–	–	1,285	22.4

^a^ Dashes demonstrate data that was not collected in the 2019 survey.

Among mothers who provided responses to birth locations in 2019, 2,244 (86.3%) reported a facility childbirth as compared to 356 (13.7%) home births, while in 2021, 5,544 (95.7%) mothers reported a facility childbirth as compared to 248 (4.3%) home births (Table 3). Additionally, in 2019, 3,064 (90.0%) mothers reported SBA, and 342 (10.0%) mothers did not utilize SBA (Table 4). In 2021, 5,684 (95.9%) mothers cited SBA as compared to 244 (4.1%) without (Table 4). Notably, in 2019, only 20 mothers delivered in a facility without SBA (0.9% of reported facility childbirths), while 46 mothers delivered outside of a facility with SBA (13.2% of home childbirths). Similarly, in 2021, only 19 mothers delivered in a facility without SBA (0.4% of facility childbirths), while 36 mothers delivered outside of a facility with SBA (14.6% of home childbirths). A total of 303 mothers (7.9% of those surveyed) delivered at home without SBA in 2019, while a total of 210 mothers (3.6% of those surveyed) delivered at home without SBA in 2021.

**Table 3:** Prevalence of facility childbirths in 2019 and 2021.

Variables	2019				2021			
	Facility (*n* = 2,244)		Home (*n* = 356)		Facility (*n* = 5,544)		Home (*n* = 248)	
	*n* (%)	95% CI	*n* (%)	95% CI	*n* (%)	95% CI	*n* (%)	95% CI
Overall	2,244 (86.3)	84.9–87.6	356 (13.7)	12.4–15.1	5,496 (95.7)	95.2–96.2	246 (4.3)	3.8–4.8
Place of residence								
NK	120 (93.8)	88.0–96.9	8 (6.3)	3.1–12.0	684 (97.9)	96.5–98.7	15 (2.2)	1.3–3.5
EK	216 (82.1)	77.0–86.3	47 (17.9)	13.7–23.0	701 (97.1)	95.5–98.1	21 (2.9)	1.9–4.4
CK	736 (94.0)	92.1–95.5	47 (6.0)	4.5–7.9	732 (96.6)	95.0–97.7	26 (3.4)	2.3–5.0
SK	581 (83.1)	80.2–85.7	118 (16.9)	14.3–19.8	678 (95.5)	93.7–96.8	32 (4.5)	3.2–6.3
CKK	291 (83.1)	78.8–86.3	59 (16.9	13.3–21.2	667 (94.1)	92.1–95.6	42 (5.9)	4.4–7.9
WK	300 (79.6)	75.2–83.4	77 (20.4)	16.6–24.8	699 (92.1)	89.9–93.8	60 (7.9)	6.2–10.1
NS	–	–	–	–	716 (95.3)	93.6–96.6	35 (4.7)	3.4–6.4
CS	–	–	–	–	667 (97.5)	96.0–98.5	17 (2.5)	1.5–4.0
Mother's age at birth								
≤20	–	–	–	–	1,118 (96.0)	94.7–97.0	47 (4.0)	3.0–5.3
21–30	–	–	–	–	3,166 (96.5)	95.8–97.0	116 (3.5)	3.0–4.2
31–40	–	–	–	–	968 (94.6)	93.1–95.9	55 (5.4)	4.1–6.9
41–50	–	–	–	–	165 (88.7)	83.3–92.5	21 (11.3)	7.5–16.7
>50	–	–	–	–	79 (91.9)	83.8–96.1	7 (8.1)	3.9–16.2
Education level								
Primary or less	1,236 (80.7)	78.6–82.6	296 (19.3)	17.4–21.4	2,878 (94.1)	93.2–94.9	180 (5.9)	5.1–6.8
Secondary or more	1,008 (94.4)	92.8–95.6	60 (5.6)	4.4–7.2	2,666 (97.5)	96.9–98.0	68 (2.5)	2.0–3.1
Marital status								
Married/Cohabiting	1,753 (87.4)	85.9–88.8	252 (12.6)	11.2–14.1	4,920 (96.1)	95.5–96.6	200 (3.9)	3.4–4.5
Unmarried	490 (82.5)	79.2–85.3	104 (17.5)	14.7–20.8	624 (92.9)	90.6–94.6	48 (7.1)	5.4–9.4
Religion								
Catholic	347 (88.3)	84.7–91.1	46 (11.7)	8.9–15.3	871 (96.0)	94.5–97.1	36 (4.0)	2.9–5.4
7th-Day Adventist	915 (90.4)	88.4–92.1	97 (9.6)	7.9–11.6	2,286 (97.0)	96.3–97.6	70 (3.0)	2.4–3.7
Protestant	488 (83.0)	79.7–85.8	100 (17.0)	14.2–20.3	1,127 (95.3)	94.0–96.4	55 (4.7)	3.6–6.0
Roho Church	363 (81.4)	77.5–84.7	83 (18.6)	15.3–22.5	898 (93.2)	91.4–94.6	66 (6.8)	5.4–8.6
Other	102 (83.6)	75.9–89.2	20 (16.4)	10.8–24.1	362 (94.5)	91.7–96.4	21 (5.5)	3.6–8.3
ANC visits								
<4	712 (81.9)	79.2–84.4	157 (18.1)	15.6–20.8	1,090 (90.7)	88.9–92.2	112 (9.3)	7.8–11.1
≥4	1,479 (89.2)	87.6–90.6	179 (10.8)	9.4–12.4	4,454 (97.0)	96.5–97.5	136 (3.0)	2.5–3.5
CHW visitation								
Yes	–	–	–	–	1,990 (96.1)	95.2–96.9	80 (3.9)	3.1–4.8
No	–	–	–	–	270 (96.8)	93.9–98.3	9 (3.2)	1.7–6.1
Wealth Tertile								
Poor	709 (78.3)	75.4–80.8	197 (21.7)	19.2–24.6	1,819 (93.9)	92.8–94.9	118 (6.1)	5.1–7.2
Middle	736 (87.9)	85.5–90.0	101 (12.1)	10.0–14.5	1,834 (95.6)	94.6–96.4	85 (4.4)	3.6–5.4
Rich	786 (93.5)	91.6–94.9	55 (6.5)	5.0–8.4	1,865 (97.7)	96.9–98.3	44 (2.3)	1.7–3.1
Parity								
1	–	–	–	–	1,719 (96.9)	96.0–97.6	55 (3.1)	2.4–4.0
2–3	–	–	–	–	2,437(95.9)	95.1–96.6	103(4.1)	3.4–4.9
4+	–	–	–	–	1,161(93.0)	91.4–94.2	88(7.1)	5.8–8.6

^a^ Prevalence of facility vs. home childbirths by explanatory variable across the 2019 and 2021 datasets.

^b^ Dashes demonstrate data that was not collected in the 2019 survey.

**Table 4:** Prevalence of skilled birth attendance in 2019 and 2021.

Variables	Skilled birth attendance							
	2019				2021			
	Yes (*n* = 3,064)		No (*n* = 342)		Yes (*n* = 5,684)		No (*n* = 244)	
	*n* (%)	95% CI	*n* (%)	95% CI	*n* (%)	95% CI	*n* (%)	95% CI
Overall	3,064 (90.0)	88.9–90.9	342 (10.0)	9.0–11.1	5,684 (95.9)	95.3–96.4	244 (4.1)	3.6–4.7
Place of residence								
NK	148 (95.5)	93.6–96.4	7 (4.5)	3.6–6.4	717 (98.2)	97.0–99.0	13 (1.8)	1.0–3.0
EK	401 (89.3)	85.7–91.5	48 (10.7)	8.5–14.3	731 (97.7)	96.4–98.6	17 (2.3)	1.4–3.6
SK	813 (87.4)	86.1–91.9	117 (12.6)	8.1–13.9	705 (95.8)	94.1–97.0	31 (4.2)	3.0–5.9
CK	928 (95.2)	90.8–97.8	47 (4.8)	2.2–9.2	705 (96.4)	94.8–97.5	28 (3.6)	2.5–5.2
CKK	402 (88.9)	85.1–89.4	50 (11.1)	10.6–14.9	687 (94.5)	92.6–95.9	40 (5.5)	4.1–7.4
WK	372 (83.6)	79.9–86.8	73 (16.4)	13.2–20.1	705 (91.6)	89.4–93.3	65 (8.4)	6.7–10.6
NS	–	–	–	–	725 (95.7)	93.9–96.9	33 (4.4)	3.1–6.1
CS	–	–	–	–	664 (97.5)	96.0–98.4	17 (2.5)	1.6–4.0
Mother's age								
≤20	–	–	–	–	1,168 (96.4)	95.2–97.3	44 (3.6)	2.7–4.8
21–30	–	–	–	–	3,262 (96.5)	95.8–97.1	118 (3.5)	2.9–4.2
31–40	–	–	–	–	996 (94.7)	93.1–95.9	56 (5.3)	4.1–6.9
41–50	–	–	–	–	174 (89.7)	84.5–93.3	20 (10.3)	6.7–15.5
>50	–	–	–	–	84 (93.3)	85.9–97.0	6 (6.7)	3.0–14.1
Education level								
Primary or less	1,676 (85.7)	84.1–87.2	280 (14.3)	12.8–15.9	2,946 (94.2)	93.3–95.0	181 (5.8)	5.0–6.6
Secondary or more	1,386 (95.7)	94.5–96.6	62 (4.3)	3.4–5.5	2,738 (97.8)	97.1–98.2	63 (2.3)	1.8–2.9
Marital status								
Married/Cohabiting	2,401 (90.9)	89.7–91.9	241 (9.1)	8.1–10.3	5,039 (96.2)	95.6–96.7	200 (3.8)	3.3–4.4
Unmarried	662 (86.8)	84.2–89.0	101 (13.2)	11.0–15.8	645 (93.6)	91.5–95.2	44 (6.4)	4.8–8.5
Religion								
Catholic	471 (91.3)	88.5–93.4	45 (8.7)	6.6–11.5	895 (96.7)	95.3–97.6	31 (3.4)	2.4–4.7
7th-Day Adventist	1,243 (92.8)	91.3–94.1	96 (7.2)	5.9–8.7	2,325 (97.1)	96.3–97.7	70 (2.9)	2.3–3.7
Protestant	687 (87.3)	84.8–89.4	100 (12.7)	10.6–15.2	1,157 (95.2)	93.9–96.3	58 (4.8)	3.7–6.1
Roho Church	481 (86.8)	83.7–89.4	73 (13.1)	10.6–16.3	936 (93.7)	92.0–95.0	63 (6.3)	5.0–8.0
Other	141 (88.1)	82.1–92.3	19 (11.9)	7.7–17.9	371 (94.4)	91.6–96.3	22 (5.6)	3.7–8.4
ANC visits								
<4	949 (86.5)	84.4–88.4	148 (13.5)	11.6–15.6	1,128 (91.8)	90.1–93.2	101 (8.2)	6.8–9.9
≥4	2,038 (92.1)	90.9–93.1	175 (7.9)	6.9–9.1	4,556 (97.0)	96.4–97.4	143 (3.0)	2.6–3.6
CHW visitation								
Yes	–	–	–	–	2,051 (96.3)	95.4–97.0	79 (3.7)	3.0–4.6
No	–	–	–	–	285 (97.3)	94.6–98.6	8 (2.7)	1.4–5.4
Wealth tertile								
Poor	953 (83.4)	81.1–85.4	190 (16.6)	14.6–18.9	1,857 (94.4)	93.3–95.3	110 (5.6)	4.7–6.7
Middle	1,001 (90.9)	89.1–92.5	100 (9.1)	7.5–10.9	1,889 (95.7)	94.7–96.5	85 (4.3)	3.5–5.3
Rich	1,090 (95.5)	94.2–96.6	51 (4.5)	3.4–5.8	1,912 (97.6)	96.8–98.1	48 (2.5)	1.9–3.2
Parity								
1	–	–	–	–	1,792 (97.0)	96.1–97.7	56 (3.0)	2.3–3.9
2–3	–	–	–	–	2,517(96.2)	95.4–96.9	99(3.8)	3.1–4.6
4+	–	–	–	–	1,196(93.1)	91.5–94.3	89(6.9)	5.7–8.5

^a^ Prevalence of skilled birth attendance by explanatory variable across 2019 and 2021 datasets.

^b^ Dashes demonstrate data that was not collected in the 2019 survey.

### Facility childbirth

The overall prevalence of facility childbirths in survey respondents was 86.3% (95% CI 84.9–87.6) in 2019 and 95.7% (95% CI 95.2–96.2) in 2021 (Table 3). In 2019, the highest rate of facility childbirth was in Central Kamagambo (94.0%, 95% CI 92.1–95.5) followed by 93.8% (95% CI 88.0–96.9) in North Kamagambo. In 2021, the highest rate of facility childbirth was in North Kamagambo (97.9%, 95% CI 96.5–98.7). In both 2019 and 2021, the lowest rates of facility childbirths were in West Kanyamkago (79.6%, 95% CI 75.2–83.4, and 92.1%, 95% CI 89.9–93.8, respectively). Home births were more common among women who were over 40 years of age at birth, in the poor wealth tertile, had only primary education, were unmarried, had less than four ANC visits, and had higher parity.

### Skilled birth attendance

The overall prevalence of SBA increased from 90.0% (95% CI 88.9–90.9) in 2019 to 95.9% (95% CI 95.3–96.4) in 2021 (Table 4). The highest rates of SBA for both 2019 and 2021 occurred in North Kamagambo (95.5%, 95% CI 93.6–96.4, and 98.2%, 95% CI 97.0–99.0, respectively). The lowest rates for both years occurred in West Kanyamkago (83.6%, 95% CI 79.9–86.8, and 91.6%, 95% CI 89.4–93.3, respectively).

As only 20 mothers (0.9% of facility childbirths) and 19 mothers (0.4% of facility childbirths) delivered in a facility without SBA in 2019 and 2021, respectively, just one regression analysis was conducted using the SBA data. Respondents from North Kamagambo had significantly more SBA compared to any other ward (Table 5). Mothers who had primary education or less were less likely to report SBA at birth than mothers with secondary education or more (APR = 0.97, *p* = 0.002). Mothers who were unmarried were also less likely to report SBA at birth as compared to those who were married or cohabiting (APR = 0.97, *p* = 0.002). Mothers who attended at least four ANC visits were 1.04 times more likely to report SBA at birth than mothers who reported less than four ANC visits (APR = 1.04, *p* = 0.002). Additionally, mothers in the “middle” and “rich” wealth tertiles were more likely to report SBA than those in the “poor” wealth tertile (APR = 1.02 and 1.03, *p* = 0.001 and <0.001, respectively). Mother's religion was not significantly associated with SBA.

**Table 5:** Factors associated with skilled birth attendance and longitudinal change from 2019 to 2021.

Variables	APR (95% CI)	*p*-value	Trend prevalence ratio (95% CI)	*p*-value
Year				
2019	REF	REF	–	–
2021	1.05 (1.03–1.06)	**<0**.**001**	–	–
Place of residence				
NK	REF	REF	1.02 (0.98–1.06)	0.291
EK	0.97 (0.96–0.99)	**0**.**008**	1.07 (1.04–1.11)	**<0**.**001**
SK	0.95 (0.93–0.97)	**<0**.**001**	1.00 (0.98–1.02)	0.892
CK	0.98 (0.96–0.99)	**0**.**031**	1.08 (1.05–1.11)	**<0**.**001**
CKK	0.96 (0.94–0.98)	**<0**.**001**	1.05 (1.01–1.08)	**0**.**019**
WK	0.93 (0.91–0.95)	**<0**.**001**	1.06 (1.01–1.12)	**0**.**015**
Education level				
Primary or less	0.96 (0.95–0.97)	**<0**.**001**	1.07 (1.05–1.10)	**<0**.**001**
Secondary or more	REF	REF	1.02 (1.00–1.03)	**0**.**012**
Marital status				
Married/Cohabiting	REF	REF	1.05 (1.03–1.06)	**<0**.**001**
Unmarried	0.97 (0.96–0.99)	**0**.**002**	1.06 (1.02–1.09)	**0**.**003**
Religion				
Catholic	1.02 (0.99–1.05)	0.152	1.03 (1.01–1.07)	**0**.**020**
Seventh-Day Adventist	1.01 (0.99–1.04)	0.299	1.03 (1.02–1.05)	**<0**.**001**
Protestant	1.00 (0.97–1.03)	0.983	1.07 (1.04–1.11)	**<0**.**001**
Roho Church	0.99 (0.97–1.02)	0.766	1.06 (1.02–1.10)	**0**.**004**
Other	REF	REF	1.05 (0.98–1.12)	0.189
ANC visits				
<4	REF	REF	1.04 (1.01–1.07)	**0**.**008**
≥4	1.04 (1.03–1.06)	**<0**.**001**	1.05 (1.03–1.06)	**<0**.**001**
Wealth tertile				
Poor	REF	REF	1.09 (1.06–1.12)	**<0**.**001**
Middle	1.02 (1.01–1.04)	**0**.**001**	1.04 (1.02–1.06)	**0**.**001**
Rich	1.03 (1.02–1.05)	**<0**.**001**	1.01(0.99–1.03)	0.122

^a^ Explanatory variables associated with skilled birth attendance according to the generalized linear model (APR is adjusted prevalence ratio).

^b^ Trend Prevalence Ratios are adjusted statistics demonstrating change over time in skilled birth attendance for each explanatory variable.

^c^ A *p*-value of <0.05 is considered statistically significant (bold).

**Figure 2:**
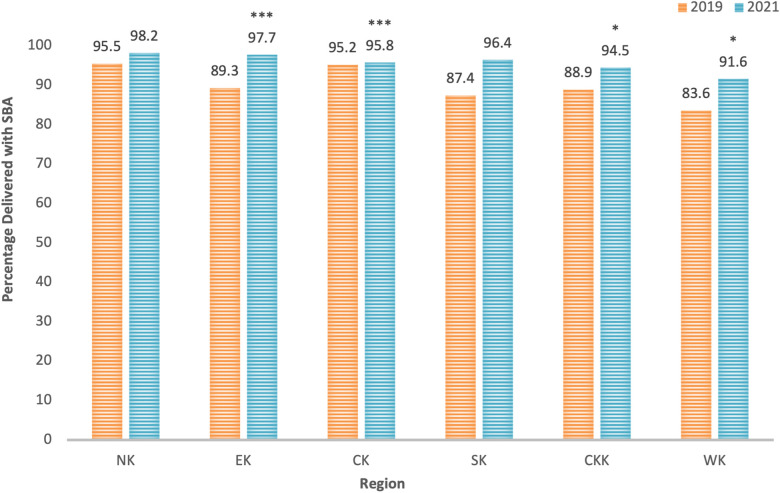
Percentage of sample reporting skilled birth attendance from 2019 to 2021 by ward. Wards: North Kamagambo (NK), East Kamagambo (EK), Central Kamagambo (CK), South Kamagambo (SK), Central Kanyamkago (CKK), and West Kanyamkago (WK). Significance is based on Trend Prevalence Ratio *p*-values from Table 5 above. *p < 0.05, **p < 0.01, ***p < 0.001.

From 2019 to 2021, the total prevalence of SBA increased significantly from 90.0% to 95.9% (APR 1.05, *p* < 0.001) (Tables 4, 5). All wards experienced this increase in SBA, and all were significant except North and South Kamagambo (Figure 2). SBA progress was observed in all mothers, regardless of education level, marital status, religion, ANC care, or wealth (Table 5). The SBA increase in the “rich” wealth tertile, 95.5%–97.6%, was not significant (Tables 4, 5).

The DiD analysis for SBA in Lwala intervention wards compared to the non-intervention wards gave an interaction term APR of 1.05 (*p* < 0.001, 95% CI 1.03–1.08) (Table 6), indicating significantly increased SBA in Lwala intervention wards.

**Table 6:** DiD analysis for skilled birth attendance in wards with vs. without Lwala intervention.

Variable	APR (95% CI)	*p*-value
Intervention ward	0.98 (0.96–1.00)	0.078
Post Intervention	1.02 (1.01–1.04)	0.007
Intervention wards post intervention^a^	1.05 (1.03–1.08)	<0.001

^a^ Coefficients and 95% confidence intervals from a DiD regression model in which “Intervention Ward” is an indicator for whether the ward was selected for the Lwala Intervention and “Post Intervention” is an indicator for time periods after Lwala Intervention was implemented. The coefficient of interest is the interaction term on intervention wards and post intervention, which measures the change in SBA in wards that implemented Lwala intervention to wards that did not, adjusted for education level, marital status, religion, ANC, and wealth tertile.

^b^ APR is adjusted prevalence ratio.

## Discussion

We report the prevalence and predictors of facility childbirth and SBA in a large household sample living in Migori County, Kenya. We found generally higher SBA and facility childbirth rates compared to regional averages. Rates increased between 2019 and 2021 across nearly all subgroups. SBA rate increased more rapidly in wards receiving Lwala programming than in those that did not receive interventions.

Our 2021 SBA rate of 95.9% is higher than the Migori county coverage reported in the 2022 Kenya Demographic and Health Survey (DHS) (92.6%) (21). Our 2021 facility childbirth rate (95.7%) is also higher than the 2022 DHS report (89.2%) (21). This discrepancy may be partially explained by the survey primarily containing wards where Lwala programming has been implemented, while the 2022 Kenya DHS covers all of Migori County (21, 23). The trend analysis demonstrates increases in SBA in surveyed wards from 2019 to 2021. Specifically, SBA increased in East Kamagambo and South Kamagambo, and this increase was significant in East Kamagambo. These increases were expected, as in these two wards, Lwala initiatives had not begun before the completion of the 2019 survey but were implemented prior to the 2021 survey. In Central Kamagambo, where interventions were not implemented prior to the 2021 survey, and in the comparison wards, Central Kanyamkago and West Kanyamkago, significant increases in SBA were observed without Lwala programs.

To distinguish the increase in SBA rate in the intervention wards from general longitudinal improvement in the comparison wards, a DiD analysis was performed. The DiD demonstrated that the SBA increase was significantly higher in the wards receiving intervention than in the comparison wards. This result suggests that Lwala programming increased SBA rates above the temporal trends in comparison wards. The 2021 survey found that the highest rates of facility childbirth and SBA occurred in North Kamagambo, the oldest ward of Lwala intervention. All wards demonstrated significantly less SBA than NK with the combined 2019 and 2021 data, but this gap may close as interventions continue in other wards. This difference was expected in Central Kamagambo, where interventions were not implemented prior to the 2021 survey, and in Central Kanyamkago and West Kanyamkago, the two comparison wards.

Lwala programming, in partnership with Migori County government, includes various initiatives to increase the frequency and quality of skilled maternal and antenatal care (22). The community-led model aims to professionalize community health workers (CHWs), including traditional birth attendants (TBAs) (22). The 2022 Community Health Services Act promotes CHW professionalization and community-level healthcare leadership (22). Lwala supports government CHWs through compensation, training, supervision, and provision of physical and digital tools to elevate their level of care (22). Provision of such equipment is further enabled by Lwala efforts to amplify the national Electronic Community Health Information System (eCHIS) in Migori County. Given recent study findings that experience and professionalization are better indicators of CHW performance than literacy and formal education (4, 30), Lwala launched National Certification guidelines for CHWs designed to be more inclusive of varied education and literacy levels (22). TBAs had historically been prevented from achieving CHW status due to education requirements, despite their essential, established role of providing community health services (22). They are now increasingly registered and certified as CHWs in Migori County using a TBA assessment tool developed by Lwala (22). A 2023 study found that clients of Lwala-trained CHWs were 14% more likely to receive four or more ANC visits (30), which we found to be a significant predictor of SBA. Therefore, Lwala interventions may contribute to improved maternal and antenatal care through a multitude of pathways.

Previous studies concur that CHWs play an essential role in improving overall health outcomes and care-seeking behavior (31–34). Furthermore, CHWs have been found to specifically improve knowledge of dangerous symptoms during pregnancy and preparedness for childbirth which in turn increases facility childbirths, further emphasizing the potential impact of Lwala initiatives (35). Lwala efforts have also improved access to healthcare by building new health facilities and maternity wards which likely also contributed to the increase in facility childbirths (22). While we did not find CHW visitation to be a significant predictor of SBA in 2021, this may be due to a disproportionately small portion of the 2021 sample that did not receive CHW visitation (12.1%) and the limitation that CHW visitation in the three months prior to the survey may not have coincided with the mother's pregnancy or childbirth.

Several explanatory variables used in our model demonstrated a significant association with SBA. As expected, higher educational status, at least four ANC visits, marriage/cohabitation, and wealth were significantly associated with SBA. Women with higher education levels are typically more likely to utilize SBA services during childbirth (36–39). This association may be observed due to the capacity that an educated woman has to locate and understand information regarding maternal health services and signs of obstetric danger, allowing them to seek care immediately when necessary (40, 41). Prior research has also found an association between ANC and both SBA and facility childbirth (42–44). Consistent ANC allows for a smooth transition into skilled childbirth as well as other necessary health services (44). Comparable to our study, other studies concur that women who are married and more wealthy are more likely to report SBA (41–45). This association may be attributed to the increased socioeconomic status and social support that is associated with marriage and partnership (46–48). Furthermore, higher economic status is typically associated with both higher use of SBA and rates of facility childbirth (37, 42). The cost of delivery services has historically been a barrier to SBA in low and middle-income countries (49). To address this barrier, the government of Kenya implemented free maternal services in 2013 which increased the national prevalence of facility childbirths (50). However, the provision of free services only addresses one aspect of financial accessibility (51, 52). The indirect transportation costs and loss of employment earnings may be equally devastating and contribute to low utilization of facilities (49, 51, 53). Lwala's continued efforts to provide more geographically accessible services have the potential to continue mitigating these challenges. The trend analysis from 2019 to 2021 displayed significant increases in SBA in the poor and middle tertiles, while the increase in the rich tertile was not significant. This finding may demonstrate progress towards equity in accessibility for families of lower socioeconomic statuses.

### Strengths and limitations

This study presents the first quasi-experimental evidence of improved SBA through Lwala Community Alliance intervention. Given the clear breakdown of Lwala initiatives by geographic ward, nearby comparison areas allow for analysis despite simultaneous temporal improvement in SBA. In 2022, SBA in Migori County surpassed averages in Kenya by 3%, sub-Saharan Africa by 13%, and worldwide by 7% (2, 21). Our findings highlight the role that Lwala may play in Migori County's improvement. Lwala programs may provide a model for SBA initiatives in similar settings, including additional sub-counties of Kenya that disproportionately bear the burden of low SBA rates and subsequent maternal and newborn mortality.

The main limitation of this study is that it utilizes a cross-sectional survey which relies on respondent recall. This also means that the explanatory variables, such as marital status, wealth, and education, are reported at the time of the survey, while the childbirth may have occurred during different, past life circumstances. The survey also relies on respondents to accurately remember the status of their childbirths in terms of SBA and location. However, given that birth of a child is a major life event, it is unlikely that the respondents would misremember the location or presence of SBA, so this is unlikely to affect the results. Finally, the generalizability of Lwala programming is not fully established due to limited implementation of interventions to certain geographic areas. Future studies including larger geographic areas and more timepoints are needed to further characterize these findings.

## Conclusions

Our cross-sectional analysis indicates that Lwala interventions are having a significant effect on increasing SBA within their wards. We describe prevalence of SBA and facility childbirth in Migori County, Kenya and associated factors, including wealth, marriage or cohabitation, education, and antenatal care. The rates of SBA and facility childbirth provide a baseline for continued evaluation of Lwala programs in our 10-year study design. The ward-level data highlights specific targets for improved programming, and the explanatory variables allow for broad conclusions that may benefit initiatives in similar settings.

## Data Availability

The original contributions presented in the study are included in the article/Supplementary Material, further inquiries can be directed to the corresponding author.
